# Data-Centric Multiobjective QoS-Aware Routing Protocol for Body Sensor Networks

**DOI:** 10.3390/s110100917

**Published:** 2011-01-17

**Authors:** Md. Abdur Razzaque, Choong Seon Hong, Sungwon Lee

**Affiliations:** Department of Computer Engineering, Kyung Hee University, 1 Seocheon, Giheung, Yongin, Gyeonggi, 446-701, Korea; E-Mails: m_a_razzaque@yahoo.com (M.A.R.); drsungwon@khu.ac.kr (S.L.)

**Keywords:** QoS-Aware routing, Body Sensor Networks, Lexicographic Optimization, Service differentiation, Localized routing

## Abstract

In this paper, we address Quality-of-Service (QoS)-aware routing issue for Body Sensor Networks (BSNs) in delay and reliability domains. We propose a data-centric multiobjective QoS-Aware routing protocol, called DMQoS, which facilitates the system to achieve customized QoS services for each traffic category differentiated according to the generated data types. It uses modular design architecture wherein different units operate in coordination to provide multiple QoS services. Their operation exploits geographic locations and QoS performance of the neighbor nodes and implements a localized hop-by-hop routing. Moreover, the protocol ensures (almost) a homogeneous energy dissipation rate for all routing nodes in the network through a multiobjective Lexicographic Optimization-based geographic forwarding. We have performed extensive simulations of the proposed protocol, and the results show that DMQoS has significant performance improvements over several state-of-the-art approaches.

## 1. Introduction

Wireless Body Sensor Networks (BSNs) have been receiving more and more attention in academia and industry in recent years, especially under the impending healthcare crisis and due to the availability of much less expensive biomedical sensors (BMSs) with certain computation and communication capabilities. The primary target applications of BSN research, so far, are medical healthcare services, addressing the weaknesses of traditional patient data collection system, such as imprecision (qualitative observation) and undersampling (infrequent assessment) [1,2]. BSNs can offer a paradigm shift from managing illness to proactively managing wellness by focusing on prevention and early detection/treatment of diseases, thereby reducing healthcare costs. They can capture accurate and quantitative data from a variety of sensors (e.g., temperature, blood pressure, heart rate, electrocardiogram (ECG), *etc.*) for longer time periods. BSNs with real-time sensing capability would also help in protecting those exposed to potentially life-threatening environments, including soldiers, first responders, and deep-sea and space explorers [3]. Therefore, on-time and reliable data delivery to the control center is very important for BSN applications.

The Quality-of-Service (QoS) provisioning in BSNs is a challenging task, mainly due to two reasons. First, the dynamic network topology, time-varying wireless channel and scarcity of node energy, computation power and channel bandwidth pose challenges on the design of QoS support schemes in BSNs. Second, there exist wide variations in data generation rate and delay- and loss-tolerances amongst the data packets generated by different types of BMSs [2]. For example, some low data rate BMSs (e.g., heartbeat, blood pressure, electroencephalogram (EEG) sensors) may generate very time-critical data packets, which must be delivered at the destination sink within a guaranteed end-to-end delay deadline; data packets from some of these sensors might also require high reliability. In contrast, some high data rate BMSs (e.g., streaming of ECG signals) may allow a certain percentage of packet losses. Therefore, a scalable solution with data-centric QoS-aware routing that can provide a clear differentiation in route selection between data packets with multiobjective QoS requirements, is greatly required for BSNs.

In the literature, several mechanisms (outlined in Section 2) have been proposed to mitigate the problems of multiobjective QoS provisioning in wireless sensor networks. However, to the best of our knowledge, no effective solution to this problem has yet been proposed so far particularly for BSNs. The key contribution of this paper is the first complete design and evaluation of data-centric multiobjective QoS-aware routing for BSNs that has clear differentiation in route selection between multiple traffic types with respect to their QoS requirements. It also trades off the energy cost and protocol operation overheads while improving the network performance.

The proposed data-centric multiobjective QoS-aware routing protocol, DMQoS, uses modular architecture and it exploits geographic locations to implement localized routing. An important property of the proposed protocol is the end-to-end QoS-aware routing with local decisions at each intermediate node without end-to-end path discovery and maintenance. This property is important for scalability to large-scale sensor networks, self-adaptability to network dynamics, and appropriateness to multiple classes of traffic flows. The routings of delay-critical and reliability-critical packets are handled separately by employing independent modules for each, whereas for the most critical packets having both stringent delay and reliability constraints, the corresponding modules operate in coordination to guarantee the required service. While the delay control module chooses the next-hop router node offering higher velocity of data packets, the reliability control module injects minimal redundant information by exploiting high reliability links. A Lexicographic Optimization (LO) [4] based approach is used to tune trade-off between the geographic progress and the residual energy levels. Therefore, our model considers not only the QoS requirements, but also the energy cost optimality of the routing path to improve the overall network performance.

We evaluated our routing protocol in terms of end-to-end packet delays, packet delivery ratio, average energy consumption per packet, and routing overhead for variable source traffic loads and wireless link bit error rates. The results show that DMQoS demonstrates a substantial improvement in required data delivery services over several state-of-the-art approaches and we provide insights into the sources of the improvement.

The rest of the paper is organized as follows. In Section 2, we describe the key limitations of some existing QoS-aware routing protocols. Subsequently, we present a body area sensor network model and assumptions we have considered in Section 3. Section 4 presents the proposed DMQoS architecture in detail, followed by the performance evaluations using Network Simulator-2 [5] in Section 5. Finally, we conclude the paper in Section 6.

## 2. Related Works

Major challenges and open research issues on QoS provisioning in BSNs have been presented in [6] and a cross-layer QoS framework (that spans over three layers) for biomedical sensor networks has been proposed in [7]. In EDDD [8], an energy-efficient differentiated directed diffusion mechanism has been developed that provides with service differentiation between real time and best effort traffic. However, their designs are neither scalable nor adaptive to dynamic environment. In recent years, QoS routing in location-aware wireless sensor networks has received much research interests due to its inherent characteristics of (i) being scalable to large networks, (ii) making routing decisions based on local neighborhood information, and (iii) being very adaptive under dynamic changes and mobility as only a node’s neighborhood is affected. In [9], a reinforcement learning-based routing model for BSNs is proposed that selects a QoS route via computing neighborhood node’s Q-values and position information, but does not consider energy at all. Directional Geographic Routing (DGR) [10] constructs an application-specific number of multiple disjointed paths for routing real time video communications data in wireless sensor networks. MCMP [11] uses link delay and reliability as routing decision parameters, where data packets are duplicated at source nodes by solving optimization problem. But, this approach considers neither residual energy nor progress speed. Hence, packets may get routed to a node which is highly congested and/or energy critical. MMSPEED [12] also sends duplicate packets (probabilistically) toward multiple paths and multiple reliability- and delay-bound packets are considered for QoS provisioning. However, routing in MMSPEED fully avoids energy consideration, reducing its applicability for BSNs. A hybrid geographic routing (HGR) protocol has been designed in [13] to achieve an efficient tradeoff between energy efficiency and delay performance. A reliable and energy-efficient routing protocol (REER) has been developed in [14] exploiting geographic information and cooperative communications.

The proposals in DARA [15] and LOCALMOR[16] have some similarities with our DMQoS protocol. In DARA [15], a weighted aggregate routing metric consisting of geographic progress, delay and energy is considered for supporting critical and non-critical data packets through defining long- and short-range forwarding zones, respectively. However, the use of same routing function for both the packet types deteriorates the QoS performance. In LOCALMOR [16], the routing functions have been separated for multiple packet types; however, it uses fixed number of sinks (primary and secondary sinks) and all packets are blindly duplicated toward both the sinks, making it unscalable. Also, it increases the overhead of sending too many duplicate packets.

The distinguished features of our DMQoS protocol design from those of the above mentioned works are as follows. Its routing functions are distinct for different packet types based on their QoS requirements. Its architecture is modular and the routing modules cooperate to render enhanced QoS services to different traffic classes. It uses Lexicographic Optimization (LO) for trading-off energy and service.

## 3. Network Model and Assumptions

### 

#### Network Model

**Figure 1.**
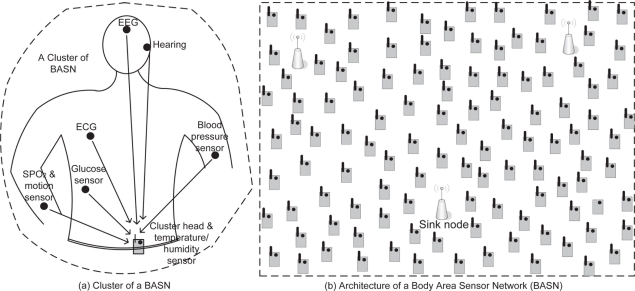
Body Sensor Network (BSN).

We assume that several biomedical sensors (BMSs) are attached to a human body, and that they acquire sensor data and transmit to a central node, namely body sensor mote (BSM), which is responsible for collecting raw data, processing (coding, aggregation, *etc.*) and forwarding them toward the sink node in multihop fashion, as shown in Figure 1. They might have one or more sensing devices as well. Note here that these central nodes, which are also considered in [16], are different from personal servers (e.g., PDA) as used in other works [17] and references therein. These sensor motes have relatively high energy and computing capability compared to tiny BMSs. We also assume that there may be several sink nodes *𝒮* in the network and any sink node *s* *∈ 𝒮* is primarily responsible for collecting and recording the patient’s data and then uploading this data to the medical care server via the Internet. In this architecture, the master BSM node and the slave BMSs together form a cluster of a body sensor network. As in Figure 1(b), a set of such cluster heads *𝒩* form the backbone of the network. The key idea used in this network architecture is to move much of the network and protocol complexity away from the power-constrained BMSs and into the much more capable cluster head node. Note that the above network architecture is not only suitable for medical healthcare applications as in [18] but also for sports, battlefield, rescue operations, *etc.*, those require large number of sensor nodes to be deployed. We also assume that each cluster head node has equal initial energy *e_init_*, and the residual energy of any node *i* *∈ 𝒩* is denoted by *e_res_*(*i*).

#### Geographic Information Consideration

In many applications of BSNs, the knowledge of the location of an event is also desired. Hence, it is important that the location of the network cluster nodes be known. If the nodes know their global or relative coordinates, distributed or stateless routing schemes can be employed, mitigating the need for propagation and updating of routing tables across the network. Distributed routing schemes based on the geographic locations of the nodes have been proposed and widely examined in [12,15] and references therein. Like them, we also assume that the coordinates of the nodes are known locally, *i.e.*, each node knows its own coordinates as well as those of its routing neighbors through execution of a HELLO protocol. All nodes would also know the coordinates of the sink(s).

#### Constraints

We assume that every delay-sensitive packet has a lifetime *t_life_*, specified by the application layer, which indicates the time limit within what the packet should be delivered to the final recipient; otherwise, the information in the packet is useless. For the reliability-sensitive packets, the application layer also tags the required reliability level *R* with a packet. Moreover, routing of all data packets should be energy-aware in order to extend the network lifetime.

#### HELLO Protocol

Each cluster head node broadcasts a HELLO packet, periodically or upon observing significant changes in some parameter values, including its *current position, residual energy* and *average queuing delays* of different packet types in the node. This HELLO protocol works in similar way as in [15,19]. Upon reception of a HELLO packet, the neighbor cluster head nodes update their neighbor table entries. A new entry is added into the neighbor table when a new node moves into vicinity, and an existing entry is deleted when a neighboring node moves away or breaks down, which can be determined when a HELLO packet is not received during a predefined period of time (timeout). Thus, HELLO packets make the neighborhood information available to each cluster head node at a cost of additional energy and bandwidth. Time between broadcasts represents a trade-off between overhead communications and outdated cost function parameters. Therefore, update frequency should be carefully chosen to maintain a proper balance between information freshness and cost.

## 4. The Proposed QoS-Aware Routing

**Figure 2.**
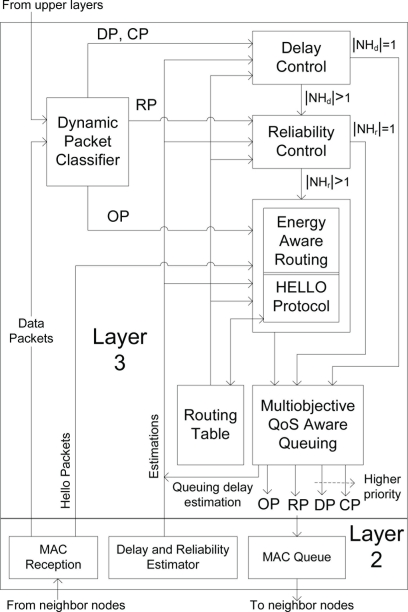
Data-centric multiobjective QoS-aware routing architecture.

Figure 2 shows the components and their interconnections in our proposed data-centric multiobjective QoS-aware routing protocol, DMQoS. The DMQoS consists of five modules: the dynamic packet classifier, delay control, reliability control, energy-aware geographic routing and QoS-aware queuing and scheduling modules. The details of these modules are presented in the following subsections.

### 4.1. Dynamic Packet Classifier

The service differentiation paradigm used in this paper is as follows. We define four classes of data packets - ordinary data packets (OP), reliability-driven data packets (RP), delay-driven data packets (DP) and most critical data packets (CP). The CP packets are given the highest priority due to their stringent delay and reliability constraints, e.g., they may carry electroencephalogram (EEG) and electrocardiogram (ECG) monitoring information during a critical situation such as a surgery. The next higher priority is given to DP packets which should be delivered within a predefined deadline, but may tolerate reasonable packet loss, e.g., video streaming. The RP packets should be delivered without loss, but do not need to be immediate or within a hard deadline, such as vital signal monitoring, respiration monitoring and PH-level monitoring. The lowest priority is given to the OP packets that are corresponding to regular measurements of patient physiological parameters, like body temperature, heartbeat, *etc.*, that typically indicate normal values.

As shown in Figure 2, on reception of data packets either from application layer or from neighbor nodes, the dynamic packet classifier (DPC) of a node assigns them to one of the proper aforementioned categories and feeds them into the respective module in first-come-first-serve (FCFS) manner.

### 4.2. Energy-Aware Geographic Forwarding

Rather than using traditional end-to-end path discovery-based routing, we use localized packet forwarding that implements hop-by-hop routing. This deferred choice gives each packet transmission multiple opportunities to make progress toward the destination.

Our goal is to select a downstream node that has comparatively higher residual energy and gives higher geographic progress toward the destination sink. Note that while the second criterion decreases the number of hops between the source and the destination, the first one attempts to balance the energy consumption among the candidate downstream nodes. Our proposed energy-aware geographic forwarding (EAGF) uses a multiobjective Lexicographic Optimization (LO) approach [4] to manage this trade-off. In LO, the objective functions are arranged according to their absolute importance and the most important objective function is maximized (or minimized) first subject to the original constraints. If this problem has a unique solution, it will solve the whole multiobjective optimization problem. Otherwise, the second most important objective function is maximized (or minimized). Now, in addition to the original constraints, a new constraint is added. This new constraint is imparted to guarantee that the most important objective function preserves its optimal value. If this problem has a unique solution, it solves the original problem; otherwise, the process goes on as above.

In solution to our problem, there are only two objective functions of which the first one, the maximization of the geographic progress, is the most important. Let the objective functions be arranged according to the lexicographic order, with the most important function being, $f_{1}(j)=\frac{\mathrm{dist}(i,s)-\mathrm{dist}(j,s)}{\mathrm{dist}(i,s)}$, ∀(*i, j*) *∈ 𝒩, ∀s ∈ 𝒮*, where *dist*(*a, b*) denotes the geometric distance between nodes *a* and *b*; and, the least important one being $f_{2}(j)=\frac{e_{\mathrm{res}}(j)}{e_{\mathrm{init}}}$. Thus, we write the lexicographic problem for any node *i* *∈ 𝒩* as follows,

(1) $$\begin{array}{ll} \text{lex maximize} & f_{1}(j),\;\;f_{2}(j)\\ \text{subject to} & \end{array}$$

(2) $$j\in {𝒩}_{i},$$

where, *𝒩_i_* is the list of *i*’s single hop neighbor nodes. The above LO problem can be divided into two separate problems with different constraint sets. The first problem is formulated as

(3) $$\begin{array}{ll} \text{maximize} & f_{1}(j)\\ \text{subject to} & \end{array}$$

(4) $$j\in {𝒩}_{i},$$

(5) $$\mathrm{dist}(i,𝒮)>\mathrm{dist}(j,𝒮),\;\;\;\forall j\in {𝒩}_{i}$$

(6) $$\mathrm{dist}(i,j)\geq \bar{\mathrm{dist}(i,j)},\;\;\;\forall j\in {𝒩}_{i}$$

and its solution $j_{1}^{*}$ and $f_{1}^{*}=\left(j_{1}^{*}\right)$ is obtained; here, $\bar{\mathrm{dist}(i,j)}$ is the average distance from node *i* to all neighbor nodes *j*. Then the second problem is formulated as

(7) $$\begin{array}{ll} \text{maximize} & f_{2}(j)\\ \text{subject to} & \end{array}$$

(8) $$j\in {𝒩}_{i}$$

(9) $$\mathrm{dist}(i,𝒮)>\mathrm{dist}(j,𝒮),\;\;\;\forall j\in {𝒩}_{i}$$

(10) $$\mathrm{dist}(i,j)\geq \bar{\mathrm{dist}(i,j)},\;\;\;\forall j\in {𝒩}_{i}$$

(11) $$e_{\mathrm{res}}(j)>\bar{e_{\mathrm{res}}(j)},\;\;\;\forall j\in {𝒩}_{i}$$

(12) $$f_{1}(j)=f_{1}^{*}$$

and the solution of this problem is $j_{2}^{*}$ and $f_{2}^{*}=\left(j_{2}^{*}\right)$; here, $\bar{e_{\mathrm{res}}(j)}$ is the average residual energy level of the neighbor nodes *j* of *i*. If $j_{2}^{*}$ does not produce a unique solution, we break the tie by selecting the one that produces the most geographic progress.

Note that the nice property of LO is its simplicity, and such a lightweight but effective method is suitable for resource-constrained BSNs. Even though LO has the drawback of neglecting less important objective functions when the most important one produces the unique solution, in our case, such situations should occur less frequently due to the high density of the network nodes. Moreover, LO exploits only local information to make routing decisions. The absence of a global routing scheme reduces the networks setup and updating costs, eliminates the need for storage of network-wide routing information at each node, and alleviates the possibility of incorrect information at the nodes as changes in system topology occur. What requires only is that the nodes must broadcast an upkeep packet including node identification, current location information, average queuing delays, and remaining energy periodically throughout its lifetime.

### 4.3. Reliability Control

Link quality degradation, congestion, node mobility, link failure(s), node failure(s), *etc.* may cause packet losses, which affect the reliable data delivery to the destination(s). It has been shown in the literature that the probability of successful packet delivery to the destination can be increased by sending duplicate packets over multiple spatially separated routes [11,12,15,16]. However, choosing the most appropriate next-hop nodes (*NH_r_*) of multiple routes toward different sinks to assure end-to-end required reliability as well as determining the optimized number of such next-hop nodes (*NH_r,opt_*) are challenging problems.

In our reliability control algorithm (see Algorithm 1), we use greedy approach to solve the above problems. At first, each node *i*, for each sink *s ∈ 𝒮*, identifies a candidate downstream node *j* that produces the maximum link reliability *r̂_i,j_* and stores it in the variable *NH_r_* (lines 1–3); the estimation method of the parameter *r̂_i,j_* will be discussed in Section 4.5. If *|NH_r_|* returns a NULL, the packet is dropped immediately; and, if it returns only one node, the packet is forwarded toward that next-hop node (lines 4–9) provided that its offered reliability is greater than the required reliability *R*; otherwise, the packet is dropped due to unavailability of feasible path. In the case *|NH_r_|* returns multiple nodes, the nodes from the list *NH_r_* are chosen one after another (in descending order of their *r̂_i,j_* values) until their aggregate reliability becomes greater than or equal to the required reliability, *R* (lines 11–17). Since the reliability is a multiplicative metric, we calculate the failure probability *F* = *F ×* (1 *− r̂_i,j_*), as presented in line 16. In fact, the above operation produces the optimal number of next-hop nodes |*NH_r,opt_|* that determines the degree of packet duplication for the reliability control. Note also that the value of *|NH_r,opt|_* ranges between 1 (best case) and the maximum number of sinks in the network (worst case). Therefore, our reliability control algorithm uses only the adequate number of duplicate packets, which in turn minimizes the routing and energy overheads compared to those of other approaches.

**Algorithm 1:** Reliability Control Algorithm, *at each source node i*.

INPUT: RP or CP packets, required reliability *R* and *i*’s single hop neighbor nodes *𝒩_i,s_*, ∀*s* ∈ *𝒮*
1. **for** each *s* ∈ *𝒮***do**
2. ${\mathrm{NH}}_{r}=\left\{j\;\in \;{𝒩}_{i,s}:{\hat{r}}_{i,j}=\underset{j\in {𝒩}_{i,s}}{\text{max}}\left({\hat{r}}_{i,j}\right)\right\}$
3. **end for**
4. **if** (|*NH_r_*| == Null) **then**
5. Drop the packet immediately;
6. **else**
7. **if** (|*NH_r_*| == 1) **then**
8. **if** (*r̂_i,j_* ≥ *R*, *j* ∈ *NH_r_*) **then**
9. *j* ∈ *NH_r_* is the desired next hop node and send the packet to the outgoing queue;
10. **else**
11. Drop the packet immediately;
12. **end if**
13. **else**
14. Sort *NH_r_* in descending order of *r̂_i,j_*
15. *NH_r,opt_* = first *j* ∈ *NH_r_*
16. *F* = (1 − *r̂_i,j_*), first *j* ∈ *NH_r_*
17. **while** (1 − *F* < *R*) **do**
18. Add next *j* ∈ *NH_r_* to the set *NH_r,opt_*;
19. *F* = F × (1 − *r̂_i,j_*)
20. **end while**
21. Call EAGF with *NH_r,opt_* as input instead of *𝒩_i,s_*;
22. **end if**
23. **end if**

### 4.4. Delay Control

This unit concerns the routing strategy for on-time delivery of time critical emergency packets. The delay-guaranteed service defines the maximum allowable latency, bounded by the lifetime of a packet (*t_life_*), required by the application. The total latency is experienced by a packet to traverse the network nodes from the source to the destination. At the network layer, the end-to-end packet latency is the sum of the processing delay, the transmission delay, the queuing delay, and the propagation delay. The queuing delay contributes most significantly to the total latency followed by the transmission delay; the other delays are negligible. The CP and DP packets should travel through the next-hop nodes that provide with higher speeds so that the delay-guaranteed service is maintained.

Our delay control algorithm is presented in Algorithm 2 that works as follows. Each node *i* first computes the required velocity of a packet, $v_{\mathrm{req}}(s)=\frac{\mathrm{dist}(i,s)}{t_{\mathrm{life}}[\mathrm{packet.type}]}$, toward any sink *s* *∈ 𝒮* based on its distance from the sink, *dist*(*i, s*), and the remaining lifetime of the packet, *t_life_*[*packet.type*]. As a packet travels, intermediate nodes update the packet’s remaining lifetime as follows, *t_life_* = *t_life_−t_elapsed_*, where *t_elapsed_* is the elapsed time of the packet at an intermediate node. We measure the *elapsed time* at each node *i* and piggyback it to the packet so that the following node *j* can determine the remaining time to deadline without using a globally synchronized clock. For this, when a node *i* receives the last bit of a packet, its MAC layer tags *t_arrival_* to the packet. This packet is processed by the network layer and forwarded to the chosen next-hop node *j* via the MAC layer. Note that the MAC layer of *i* requires some time to capture the channel using an RTS/CTS handshake and may transmit the packet several times until receiving ACK from *j*. For *i* to piggyback the accurate elapsed time, the MAC layer updates the field of elapsed time *t_elapsed_* just before it actually transmits the packet to the physical link as follows, *t_elapsed_* = *t_departure_* + *t_transDelay_ − t_arrival_*, where *t_departure_* is the time at which node *i* transmits the first bit of the packet to the physical link and *t_transDelay_* is the transmission delay of the packet which can be computed using the transmission rate and packet length. Thus, once node *j* successfully receives the packet, the packet contains the correct measurement of the elapsed time at node *i* and computes the remaining lifetime of the packet.

After that the node *i* calculates the velocities offered by candidate next-hop nodes *j* toward a sink *s ∈ 𝒮*, *v_j_*(*s*), by taking into account the packet’s waiting time at the queue of node $i,{\hat{d}}_{q}^{i}[\mathrm{packet.type}]$, the estimated packet transmission delay of node $i,{\hat{d}}_{\mathrm{tr}}^{i}$, and the packet’s waiting time at the candidate next-hop node *j*, ${\hat{d}}_{q}^{j}[\mathrm{packet.type}]$ (see instruction 4). The consideration of queuing delay at a candidate next-hop node is very important for localized and delay-constrained routing because it indicates the traffic forwarding efficiency of the node. Nodes in the congested (or high traffic) area may have higher queuing and transmission delays, and thus their offered velocities will be lower. Hence, they will not be able to meet the required velocity level of the CP or DP data packets.

For all sinks, after computing the velocities of all candidate nodes, the algorithm then identifies the set of next-hop nodes *NH_d_* that are supposed to meet the required delay deadline (see instruction 6). Note here that there could be several non-overlapping paths from a source to the destination(s) even though they may not be the shortest paths. A non-shortest path is acceptable as long as it can deliver a packet within its end-to-end delay deadline. The estimation methods for queuing and transmission delays will be discussed in Section 4.5.

In the case in which *|NH_d_|* returns a NULL value, *i.e.*, there is no neighboring router node satisfying the required velocity level, the packet is dropped immediately in order to prevent the network nodes from expending unnecessary energy. If *|NH_d_|* returns only one node, the packet is sent to the outgoing queue putting that node as the next-hop. On the other hand, if *|NH_d_|* includes several nodes, either the EAGF or the reliability control algorithm is called depending on the packet type in order to select the most appropriate router(s) from this set.

**Algorithm 2:** Delay Control Algorithm, *at each node i*.

INPUT: DP or CP packets, *i*’s single hop neighbor nodes *𝒩_i,s_*,, ∀*s* ∈ *𝒮*
1. **for** each *s* ∈ *𝒮***do**
2. Required velocity, $v_{\mathrm{req}}(s)=\frac{\mathrm{dist}(i,s)}{t_{\mathrm{life}}[\mathrm{packet.type}]}$
3. **for** each *j* ∈ *𝒩_i,s_***do**
4. Offered velocity, $v_{j}(s)=\frac{\mathrm{dist}(i,s)-\mathrm{dist}(i,j)}{{\hat{d}}_{q}^{i}[\mathrm{packet.type}]+{\hat{d}}_{\mathrm{tr}}^{i}+{\hat{d}}_{q}^{j}[\mathrm{packet.type}]}$
5. **end for**
6. *NH_d_* = {*j* ∈ *𝒩_i,s_* : *v_j_*(*s*) ≥ *v_req_*(*s*)}
7. **end for**
8. **if** (|*NH_d_*| == Null) **then**
9. Drop the packet immediately;
10. **else**
11. **if** (|*NH_d_*| == 1) **then**
12. *j* ∈ *NH_d_* is the desired next hop node and send the packet to the outgoing queue;
13. **else**
14. **if** (*packet.type* == *DP*) **then**
15. Call EAGF with *NH_d_* as input instead of *𝒩_i,s_*;
16. **else**
17. **if** (*packet.type* == *CP*) **then**
18. Call reliability control algorithm with *NH_d_* as input;
19. **end if**
20. **end if**
21. **end if**
22. **end if**

### 4.5. Parameter Estimations

#### Estimation of Queuing Delay

As shown in Figure 2, several queues are used for storing the different classes of data packets. The transmission scheduling of data packets from different queues is also different, to be discussed soon, and therefore, each class of data packets from a single node *i* may experience separate estimation of queuing delay $d_{q}^{i}$. We calculate this delay as the time difference between the insertion time of the packet at the queuing system and the time at which it enters into the position of transmission. Note here that we omitted the processing time of a packet at the node because it is trivial and is assumed to be almost the same for all packet types.

An Exponentially-Weighted Moving Average (EWMA) formula may suffice to estimate the running average queuing delays for each packet type ${\hat{d}}_{q}^{i}[\mathrm{packet.type}]$. Algorithm 3 shows the steps for estimating queuing delays, wherein, the weighting for each older data point decreases exponentially, giving much more importance to recent observations $d_{q}^{i}[\mathrm{packet.type}]$. The degree of weighing decrease is expressed as a constant smoothing factor *α*, a number usually between 0 and 0.4. In our simulation, we use *α* = 0.2.

**Algorithm 3:** Queuing Delay Estimator, *at each node i*.

1. **Initialization:**${\hat{d}}_{q}^{i}[\mathrm{packet.type}]=0$
2. **for** each packet transmission **do**
3. **if**${\hat{d}}_{q}^{i}[\mathrm{packet.type}]=0$**then**
4. ${\hat{d}}_{q}^{i}[\mathrm{packet.type}]=d_{q}^{i}[\mathrm{packet.type}]$
5. **else**
6. ${\hat{d}}_{q}^{i}[\mathrm{packet.type}]=(1-\alpha ){\hat{d}}_{q}^{i}[\mathrm{packet.type}]+{\alpha d}_{q}^{i}[\mathrm{packet.type}]$
7. **end if**
8. **end for**

#### Estimation of Transmission Delay

Each node *i ∈ 𝒩* in the MAC layer measures the average packet transmission delay ${\hat{d}}_{\mathrm{tr}}^{i}$ from itself using a Weighted Average Transmission Delay (WATD) method. WATD is very similar to the WALI method [20], which measures packet loss intervals for TCP congestion control. The WATD that works as follows. It measures the instantaneous delay for each packet transmission, $d_{\mathrm{tr}}^{i}(n)$, which is the delay for the *n^th^* packet transmission measured as the time duration from the time at which the packet is ready for transmission (becoming the head of the transmission queue) to the time of successful transfer of its last bit and calculates the average value for the last *P* packets using (13),

(13) $${\hat{d}}_{\mathrm{tr}}^{i}=\frac{\sum_{n=1}^{P}d_{\mathrm{tr}}^{i}(n)\times w_{n}}{\sum_{n=1}^{P}w_{n}}$$

where,

$$w_{n}=1-\frac{n-P/2}{P/2+1},\;\;\;P/2<n\leq P$$

For *P* = 8, this gives weights of 1, 1, 1, 1, 0.8, 0.6, 0.4 and 0.2 for *w*_1_ through *w*_8_, respectively.

Note that $d_{\mathrm{tr}}^{i}$ includes all delays due to media contention, such as channel sensing, RTS/CTS if any (depending on the chosen MAC protocol), backoff time slots, retransmissions, *etc.* Also note that the sensitivity of the average value ${\hat{d}}_{\mathrm{tr}}^{i}$ depends on the value of *P*. In practice, a value of *P* = 8, with the most recent four samples equally weighted, appears to be a lower bound that still achieves a reasonable balance between resilience to link variations and fast response to real changes in the network conditions.

#### Estimation of Link Reliability

Each node *i ∈ 𝒩* in the MAC layer also measures the average link reliability *r̂_i,j_* separately for all of the downstream nodes *j ∈ 𝒟_i_* using Windowed Mean EWMA (WMEWMA), which is very similar to EWMA but updates the estimated parameter in regular time intervals instead of doing it for every packet. This method is more appropriate for measuring link reliability. WMEWMA counts the total number of transmission attempts *TxCounter* required (including the retransmissions) to send all of the packets of the current window *W* and the number of packets successfully transmitted *SucCounter* of the same window. Therefore, the ratio $\frac{\mathrm{SucCounter}}{\mathrm{TxCounter}}$ represents the success probability of the link for the window, which we regard as the link reliability in this paper. This per window link reliability is then averaged with the previous measurements using EWMA as follows,

(14) $${\hat{r}}_{i,j}=(1-\beta )\times {\hat{r}}_{i,j}+\beta \times \frac{\mathrm{SucCounter}}{\mathrm{TxCounter}}$$

where *β* is the moving average smoothing factor, and its value is set equal to 0.4 in our simulation; a higher value is chosen because the current measurement is performed for all of the packets in a window *W* = 8 instead of for a single packet.

### 4.6. QoS-Aware Queuing and Scheduling

Queuing and scheduling have a direct impact on QoS characteristics. The desired QoS-aware routing for multiple classes of traffic, ranging from most critical data packets having constraints on both the delay and reliability to ordinary packets having no such delay or reliability constraints, is assumed to be met by implementing priority queues. In this work, four separate queues in a sensor node are considered; the highest priority queue for the critical packets (CP), the second higher priority queue for the delay-constrained packets (DP), the next lower priority queue for the reliability-constrained packets (RP) and the least priority queue for the ordinary packets (OP), as illustrated in Figure 2. Here, the scheduler uses strict priority logic, *i.e.*, it always serves the highest priority queue first. If there is no packet waiting in the higher priority queues, it will serve the lower priority queues.

A key problem of the above multi-queuing system is that the lower priority traffic may be indefinitely blocked by higher priority traffic, which is commonly known as the starvation problem. A solution to this starvation problem of the lower priority traffic is *aging*, which is a technique of gradually increasing the priorities of packets waiting in the system for a longer period of time. In this work, we use a timeout-based policy that moves a packet to an upper priority queue on expiration of the timeout. Note that the above multi-queuing system implements the in-node packet contention based on priority levels, *i.e.*, among the packets of the same node. It is also possible to define inter-node priorities for all traffic classes of the neighboring nodes by modifying the MAC protocol slots and backoff times [15]. However, the implementation of such inter-node prioritization requires important modifications in the MAC layer, which is beyond the scope of this work.

## 5. Performance Evaluation

### 5.1. Simulation Model and Method

The performance of the proposed DMQoS routing framework is studied and compared with three other localized and QoS-aware routing protocols DARA [15], LOCALMOR [16] and MMSPEED [12] using simulations based on ns-2 [5], which supports the simulation of multihop wireless networks complete with physical, data link, and MAC layer models. The configuration of network parameters is shown in Table 1. The delay and reliability constraints for each packet class are listed in Table 2. Note here that the constraints for a candidate application may vary depending upon the values generated by the sensors. For instance, BP or body temperature readings may produce CP traffic flows if the values cross certain thresholds.

For performance studies, we used five different sets of source traffic loads *S*_1_ through *S*_5_, listed in Table 3, each consisting of a certain number of traffic flows for each packet classes. For instance, traffic input set *S*_1_ consists of 5 CP traffic flows, 10 DP flows, 20 RP flows and 30 OP flows.

To prevent buffering packets indefinitely, packets are dropped if they wait in the send buffer for more than their remaining lifetimes. All packets sent via the routing layer are queued at the interface queue until the MAC layer can transmit them. The interface queue has a maximum size of 60 packets and is maintained as a priority queue with four priorities, each served in FIFO order. Routing packets get higher priority than data packets.

The average result of 10 simulation runs is calculated for each graph point. For each simulation run, the active nodes are randomly selected, and the source node for each of the flows is also randomly selected. Thus, the variations in the obtained results mainly occur due to the randomness of the topology. The error bars in the graphs parallel to the y-axis indicate the variations in the obtained results from the presented average values and, thus, show the minimum and maximum values obtained from the runs.

### 5.2. Simulation Results

We first evaluate the impacts of various traffic loads and bit error rates (BER) on the average end-to-end packet delay and the on-time packet delivery ratio. While the first parameter is measured as the average delay experienced by all classes of delivered packets to the sinks, the latter one is the ratio of the total number of packets received by the sinks to the number of packets generated by the sources.

#### Impact of Traffic Loads

**Figure 3.**
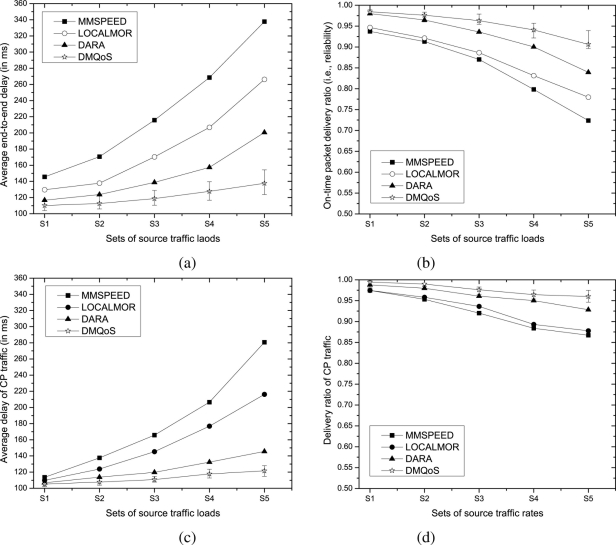
Performance comparisons for varying traffic loads- **(a)** average end-to-end delay of all data packets, **(b)** on-time packet delivery ratio *i.e.*, the achieved reliability, **(c)** average delay of CP traffic and **(d)** reliability of CP traffic.

The modular and distributed architecture of the proposed DMQoS framework helps it to achieve significant performance improvements over the state-of-the-art QoS-aware routing protocols, as shown in Figure 3. Recall (from Algorithms 1 and 2) that DMQoS optimizes the number of packet duplications based on measured link reliability (*r_i,j_*) values and chooses the next-hop router that has the highest velocity toward the destination sink. The velocity prediction of DMQoS is more accurate than those of other methods since it takes into account both transmission and queuing delays at neighbor nodes. Furthermore, the routings of different classes of data packets are handled distinctly, giving highest priority to the critical traffic flows, thus ensuring more reliable and faster delivery of important information to the destination.

As source traffic load increases, the media contention increases as well, which in turn raises packet delays at each hop toward the destination. Figure 3(a) shows that, in all approaches, the end-to-end packet delay rises to higher values with increasing traffic load. The DMQoS offers the lowest end-to-end delay, and the gaps amongst the graphs are widened as traffic volume increases, indicating that DMQoS is more capable of handling high traffic volume compared to other approaches. On the other hand, MMSPEED suffers from the highest delay followed by LOCALMOR and DARA. Through careful examination of the details of our simulation, we observe that mainly exponential increase in packet duplication in MMSPEED, blind duplication of data packets toward primary and secondary sinks in LOCALMOR and the use of the same aggregate routing function both for critical and non-critical traffic in DARA cause more contention in the wireless medium, resulting in increased end-to-end packet delivery delays.

Figure 3(b) shows that DMQoS demonstrates superior performance in terms of on-time packet delivery ratio, indicating that packet drops can greatly be reduced when source data packets are dispersed toward multiple sinks in a way that implements load-balancing among the paths by judiciously choosing the more reliable links and lightly loaded next-hop router nodes along the path. The simulation results indicate that the poor performances of LOCALMOR and MMSPEED protocols are primarily due to the excessive media contentions caused by huge numbers of duplicate packets in the networks. We also plotted the average end-to-end packet delays and delivery ratio experienced by CP traffic only in Figure 3(c) and Figure 3(d), respectively, and found that our DMQoS outperforms the existing approaches, evidencing the advantage of using the data-centric and scalable modular QoS-aware routing approach.

#### Impact of Bit Error Rate

In what follows, we investigate the impacts of wireless link bit error rates (BER) on the end-to-end packet delay and the reliability. We vary the BER value ranging from 10^−6^ to 10^−2^, keeping the data traffic load fixed at *S*_3_.

**Figure 4.**
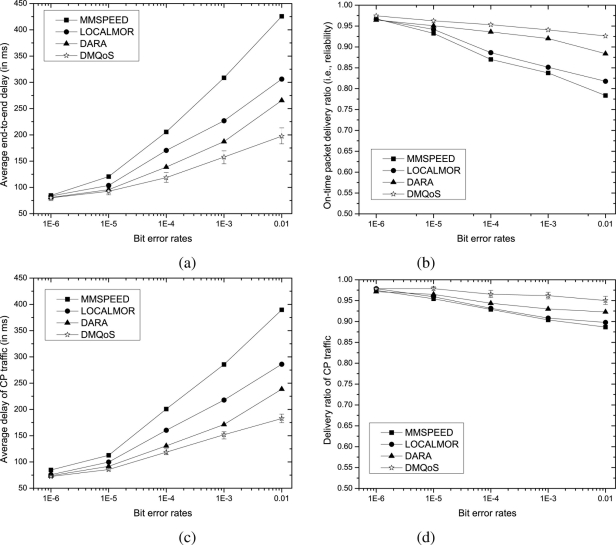
Performance comparisons for varying bit error rates- **(a)** average end-to-end delay of all data packets, **(b)** on-time packet delivery ratio, **(c)** average delay of CP traffic and **(d)** reliability of CP traffic.

With the increase of bit error rates packet delivery delay increases, and the probability of successful packet delivery decreases. More explicitly, higher value of BER raises the number of retransmissions required for each packet (at each hop) prolonging the per-hop packet delay and thereby the end-to-end delay. A packet is dropped by an intermediate node if the number of retransmissions exceeds its limit, diminishing the packet delivery ratio. However, by comparing the graphs in Figures 3 and 4, we find that the effect of bit error rate is much higher on the end-to-end delay than that on the packet delivery ratio. The reason is that, at moderate traffic loads, the contention in the wireless medium does not grow up that much, and thus, even though the packet delivery delay increases due to packet retransmissions, it can prevent the effect of bit error rates keeping the packet delivery ratio at an acceptable level.

From the results of Figure 4, we also observe that our DMQoS is more robust to the wireless link bit error rates compared to other approaches. This result is characterized by several facts: (i) DARA’s aggregate routing metric (*i.e.*, weighted-linear combination of three sub-metrics) fails to select the most appropriate next-hop node for the traffic classes, *i.e.*, it uses the same metric for CP, DP or RP traffic flows, (ii) excessive duplicate packets toward fixed number of sinks generated by LOCALMOR and MMSPEED cause high media contention and packet drops, and (iii) MMSPEED suffers most since its exponentially generated duplicated packets (along with the original packets) converge together somewhere near the single sink causing many packet drops due to collisions and buffer overflows. However, our DMQoS optimizes the number of duplicate packets, selects the most appropriate next-hop node using separate metrics for different packet classes and disperses the source traffic toward sinks offering lightly-loaded paths.

#### Impacts of Traffic Load and BER on Energy Consumption

**Figure 5.**
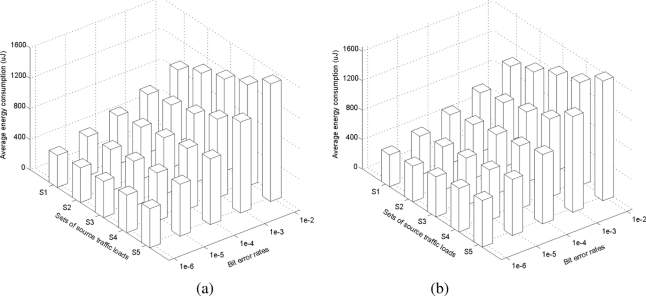
Average energy consumption per packet in **(a)** DMQoS and **(b)** DARA for varying traffic loads and bit error rates.

In this experiment, we calculate the total amount of energy consumed for transmission and reception of a packet by source and forwarder nodes until it is received by a sink and average the values for all packets. Figures 5(a) and 5(b) show the average energy consumption per packet for varying source traffic loads and bit error rates in DMQoS and DARA, respectively.

We observe that, in both approaches, the energy consumption rises up linearly for increasing source traffic loads and exponentially for increasing bit error rates. However, the rate of increase in DMQoS is slightly less than that in DARA. Our in-depth look into the simulation results reveals that this is mainly due to the reduced amount of packet collisions and retransmissions in DMQoS compared to those in DARA. As the source traffic load increases, the media contention increases as well and the data packets have to travel longer paths (for load-balancing) to reach their destinations and thus the per packet energy consumption is also increased. BER has more impacts on energy consumption since it forces packets to be retransmitted many times at each hop.

#### Protocol Operation Overhead

One further issue to consider is the amount of energy overheads due to transmission and reception of routing control packets in different approaches for varying traffic loads and bit error rates. Every QoS provisioning scheme has to exchange additional control packets (in addition to those for the basic routing mechanism) in order to update the nodes with the current neighborhood information necessary to provide better QoS services, incurring extra overhead.

**Figure 6.**
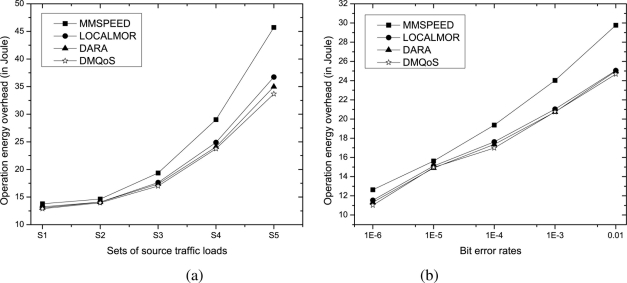
Protocol operation energy overhead due to routing control packets for **(a)** varying traffic loads and **(b)** bit error rates.

As expected theoretically, in all approaches, the amount of overhead quickly rises to higher values with increasing numbers of traffic sources. Because each source node needs to exchange routing control packets, the number of such control packets increases with the traffic load of the network as the routing parameters quickly vary. Figure 6(a) shows that MMSPEED incurs the highest overhead since it uses reliability and delay backpressure packets in addition to the neighborhood route information update messages; however, the overheads in the other three approaches are very close to each other as they all use only HELLO (or BEACON) packets to update single-hop neighborhood information. For the same reason, in Figure 6(b), we observe that the overheads in all approaches increase almost linearly with increasing bit error rates.

### 5.3. Discussions

The assembly efficiency of multiple paths is a great boon to unreliable sensor networks. Obviously, there may exist many feasible combinations. To save the energy cost, the set with minimum number of paths is chosen as the forwarding set (see Sections 4.2 and 4.3). We argue that sending a packet on more paths induces more energy cost, because more data packets have to be transmitted. Also, using more paths introduces more contentions which degrades energy efficiency. Even some paths in the set may have more hops, it is still more energy efficient to confine packets to a few paths. For the energy efficiency, as long as the delay and reliability constraints are satisfied we forward the data traffic over energy-rich nodes in order to implement an almost homogeneous energy dissipation rates for all sensor nodes in the network.

The main limitation of this paper is related to a lack of sufficient understanding about the dynamics of several estimation tuning parameters. For example, α, β and *w_n_* values were determined through numerous simulation experiments. If we could build an analytical model for them, we would be able to dynamically select the optimal values to adapt to different situations. We left it for future work.

## 6. Conclusions

Due to the overheads caused by implementing QoS in BSNs, QoS and energy must be considered together. In this paper, we have proposed a distributed flexible mechanism to optimize QoS and energy in multihop BSNs based on modular design architecture, following several traffic classes. The routing decision is localized and independent of traffic classes, which distinguishes DMQoS from other approaches. Energy is optimized among the set of candidate nodes offering data-centric QoS services (delay and/or reliability). The results signify that our data-centric multiobjective QoS-aware routing protocol provides a dynamic and modular approach for rendering quality data delivery services and is well suited to resource-constrained BSNs with comparable overhead to those of other methods.

## Figures and Tables

**Table 1.** Configuration of Parameters.

**Deployment**	Area	2,000 m × 2,000 m
	Deployment type	Random
	Number of nodes	1,000 BSMs 6,000 BMSs
	Sink locations (3 sinks)	(1000, 300) (200, 1700) (1700, 1800)
	Initial node energy	100 Joules
	Buffer size	60
	Radio range	100 m
	Link layer trans. rate	1 Mbps
	Transmit power	7.214*e*^−3^ Watt
	Rcv. signal threshold	3.65209*e*^−10^ Watt
	Bit error rate	10^−4^
**Task**	Application type	Event-driven
	Packet size	<= 32 Bytes
	Traffic type	CBR
**MAC**	IEEE 802.15.4	Default values
**Simulation**	Time	1,000 seconds

**Table 2.** QoS requirements for different applications.

**Packet Class**	**Delay constraint**	**Reliability constraint**	**Candidate applications**
CP	0.25 s	0.90	ECG, EEG
DP	0.30 s	-	Video imaging, Motion sensing, EMG
RP	-	0.95	BP, PH and respiration monitoring
OP	Only energy-aware		Glucose, SPO2, Body temperature

**Table 3.** Five different sets of source traffic loads.

Source sets	*S*_1_	*S*_2_	*S*_3_	*S*_4_	*S*_5_
Packet Class					
CP	5	10	20	30	40
DP	10	20	40	60	80
RP	20	40	60	80	100
OP	30	60	80	100	120
